# Means of Preventing the Pitting of Small Pox

**Published:** 1859-12

**Authors:** 


*Means of Preventing the Pitting of Smallpox.*—The well-known fact that the violence of the disease, in small-pox, is usually in proportion to the number of pustules, especially on the face, has led to the attempt to prevent their development by means of external applications. For this purpose, various substances which protect the skin from contact with the air have been tried, with greater or less success, such as lard, collodion and oil, or caustic solutions, especially of the nitrate of silver, and finally mercurial ointment. A writer in the *Union Medicale*, Dr. Anselmier, after examining in detail these substances, gives the preference to the last named, as being the most convenient and the most efficacious. He thinks that it has a twofold action, not only protecting the skin from the action of the air, but in consequence of its absorption, modifying the condition of the blood. Since the ointment, in its usual condition, is so soft that it is apt to penetrate between the eyelids, and irritate the conjunctiva, and also to be rubbed off by contact of the skin with the bed-clothes, he combines with it a sufficient proportion of lead plaster to give it a certain consistence. This combination becomes soft, but does not liquify, at the temperature of the skin of the face. A thin layer is to be spread over the face, neck, shoulders, arms and hands, for about a fortnight, and the effect, according to Dr. Anselmier, is to render the eruption discrete, and to prevent the papules from terminating in suppuration, in those parts to which it is applied. In case salivation should supervene, astringent gargles, with chlorate of potash, must be employed; and in such cases it is certain that there will be no eruption on the mucous membrane of the mouth.—*Boston Journal.*

